# Rapid Response to Selection, Competitive Release and Increased Transmission Potential of Artesunate-Selected *Plasmodium chabaudi* Malaria Parasites

**DOI:** 10.1371/journal.ppat.1004019

**Published:** 2014-04-24

**Authors:** Laura C. Pollitt, Silvie Huijben, Derek G. Sim, Rahel M. Salathé, Matthew J. Jones, Andrew F. Read

**Affiliations:** 1 Center for Infectious Disease Dynamics, Department of Biology, Pennsylvania State University, University Park, Pennsylvania, United States of America; 2 Centre for Immunology, Infection and Evolution, The University of Edinburgh, Edinburgh, United Kingdom; 3 Department of Entomology, The Pennsylvania State University, University Park, Pennsylvania, United States of America; 4 Fogarty International Center, National Institutes of Health, Bethesda, Maryland, United States of America; Texas Biomedical Research Institute, United States of America

## Abstract

The evolution of drug resistance, a key challenge for our ability to treat and control infections, depends on two processes: de-novo resistance mutations, and the selection for and spread of resistant mutants within a population. Understanding the factors influencing the rates of these two processes is essential for maximizing the useful lifespan of drugs and, therefore, effective disease control. For malaria parasites, artemisinin-based drugs are the frontline weapons in the fight against disease, but reports from the field of slower parasite clearance rates during drug treatment are generating concern that the useful lifespan of these drugs may be limited. Whether slower clearance rates represent true resistance, and how this provides a selective advantage for parasites is uncertain. Here, we show that *Plasmodium chabaudi* malaria parasites selected for resistance to artesunate (an artemisinin derivative) through a step-wise increase in drug dose evolved slower clearance rates extremely rapidly. In single infections, these slower clearance rates, similar to those seen in the field, provided fitness advantages to the parasite through increased overall density, recrudescence after treatment and increased transmission potential. In mixed infections, removal of susceptible parasites by drug treatment led to substantial increases in the densities and transmission potential of resistant parasites (competitive release). Our results demonstrate the double-edged sword for resistance management: in our initial selection experiments, no parasites survived aggressive chemotherapy, but after selection, the fitness advantage for resistant parasites was greatest at high drug doses. Aggressive treatment of mixed infections resulted in resistant parasites dominating the pool of gametocytes, without providing additional health benefits to hosts. Slower clearance rates can evolve rapidly and can provide a strong fitness advantage during drug treatment in both single and mixed strain infections.

## Author Summary

The evolution of drug resistance is a major challenge facing medicine in the 21^st^ century. In the case of malaria parasites, this is particularly apparent, as the introduction of each drug has been followed by the rapid development and spread of resistant parasites. Without a constant supply of new drugs to replace those that are no longer effective, it is important to understand the processes that lead to the selection and spread of resistance though a parasite population, so that the useful lifespan of current drugs can be maximized. Here, we use a rodent malaria model system to try to select for reduced susceptibility to the current frontline malaria drug, artemisinin. We then examine the growth and transmission potential of resistant parasites in single infections and in competition with susceptible parasites (mixed infections) in drug-treated hosts. We show that parasites selected for reduced susceptibility to drugs have increased fitness in both situations. Our results also indicate that the consequences of different treatment regimes on the rate of spread of resistance should be evaluated and taken into account during regime choice.

## Introduction

The evolution of drug resistance in pathogens is a major public health concern, as it has the potential to undermine many of the health advances achieved in the last century. In addition to the individual health impacts of treatment failure, drug resistance has substantial economic costs, including research into alternative treatments and new drug development [1]–[2]. In the case of malaria, the introduction of new drugs has inevitably been followed by the evolution and spread of resistance [3]–[4]. Artemisinin-based drugs (hereafter, artemisinins), the current frontline drugs against malaria parasites, are highly valued for their ability to rapidly clear infections [5]–[6]. However, in the last few years, slower parasite clearance rates, possibly an early sign of resistance, have been reported in Western Cambodia [7]–[9]. Furthermore, studies showing a genetic basis for slower clearance rates [10]–[11] and the spread of this phenotype through South East Asia [12]–[13], are causing concern that the useful lifespan of artemisinins could be limited [14]. Of particular concern is the fact that, if the currently recommended treatments fail, there are no new antimalarial drugs ready for wide-scale use [14]–[15].

Changes in the parasite response to artemisinins are unlike resistance observed to other anti-malarial drugs. Reduced clearance rates have been observed [7]–[8], and a potential genetic basis for this phenotype has been identified [10], [16], but cases of treatment failure appear rare (but see [17]). The label of ‘resistance’ is thus controversial [18]–[22]. Drug resistance is, however, often a continuous trait, with partial resistance (drug ‘tolerance’) allowing parasites to survive some drug concentrations, but increased drug doses restoring treatment efficacy [23]. ‘Full’ resistance is reached when parasites survive the highest drug dose that can be safely administered. Slower clearance rates in response to artemisinins may indicate an early, or partial, resistance phenotype, representing a stepping-stone towards full resistance [23]. For simplicity, here we use ‘resistance’ to refer to malaria parasites with reduced susceptibility to artemisinins, as judged by a reduced clearance rate during treatment.

The useful lifespan of a drug depends both on the probability that resistance arises *de novo* and on the rate of spread of resistant parasites within a population, which is in large part a function of the strength of selection. The traditional view has been that aggressive chemotherapy, involving high doses applied for sufficiently long to eliminate parasites, best minimises the evolution of resistance because it reduces the probability of a *de novo* resistant mutant arising [5], [24]. Additionally, high dose regimens will also kill off any partially-resistant parasites which may have survived less aggressive treatment [23]. However, the rate of spread of a resistant parasite is determined both by its competitive ability within individual infections and its success at transmitting through the host population [25]. Within individual hosts, aggressive treatment is predicted to exert the strongest positive selection pressure on existing resistant parasites, especially in mixed infections with susceptible competitors [26]–[27]. The majority of malaria infections consist of multiple competing genotypes and/or species [28]–[29], and resource-mediated (e.g. red blood cells), immune-mediated or, potentially, direct interference competition between strains results in suppression of parasite densities [30]–[32]. Previous work using the anti-malarial pyrimethamine has shown that removing susceptible competitors through drug treatment can lead to dramatic increases in the density of resistant parasites, termed ‘competitive release’ [27], [33]–[34]. Furthermore, different drug treatment regimes alter the magnitude of this competitive release and, therefore, the fitness of drug resistant parasites [26]: the more aggressive the drug treatment, the higher the selective advantage for resistant parasites [35]–[36]. Therefore, aggressive drug treatment can be a double-edged sword, reducing the chances of *de novo* resistance, but providing the strongest selection for resistant mutants already present in an infection [26].

The spread of the artemisinin slow-clearance phenotype in parasites in South-East Asia suggests a fitness benefit for the parasite. However, as *Plasmodium falciparum* gametocytes (the parasite stage infective to mosquitoes) take 7 days to mature [37], it is not clear how a delay in clearance of 1–2 days could result in increased transmission since the presence of gametoctyes 7 days later has not, so far as we are aware, been reported. It could be instead that slower clearing parasites are more likely to recrudesce (a secondary increase in parasite density following treatment-induced parasite clearance) [38]–[40], have increased persistence of existing transmission stages or increased competitive ability within mixed infections. Gaining a better understanding of the artemisinin resistance phenotype, and its fitness benefits, is thus essential for predicting its further spread. Evidence for competitive release of drug resistant parasites in malaria parasites has until now come from infections treated with the antimalarial drug pyrimethamine [33]–[36], [41]. Whether these findings can be generalized to other antimalarial drugs and, more specifically, to parasite lines where resistance is characterised by slower clearance rates, is unknown. In human malaria infections, host factors such as immunity, the multiplicity of infection and parasite density at the start of treatment, influences rates of parasite clearance [42]–[43]. Additionally, sensitive *in vitro* tests or molecular markers of resistance are not currently available [44]–[45], meaning that characterising parasite lines as resistant and tracking their fitness and spread is challenging. The possibility for untreated controls and limited host variation make animal models a powerful tool for examining the fitness and transmission potential of resistant parasites.

Here, we use the rodent malaria parasite *Plasmodium chabaudi* to separately examine both sides of the double-edged sword in resistance management. First, we exposed malaria parasites to various starting doses of the antimalarial artesunate, thereby testing the ability of high drug doses to prevent the origin of resistance. Second, we used one of our selected lines to examine the fitness implications of slower clearance rates and of competitive interactions between resistant and susceptible parasites under different drug pressures. This work was done across three experiments (Table 1). In experiment 1, we tested whether our selection regime led to changes in the parasite response to drugs and quantified the dose response. In experiment 2, we tested the hypothesis [8], [40], [46] that resistance to artemisinin is stage-specific by drug treating hosts at different points in the parasite life-cycle. Finally, in experiment 3, we examined the interactions between our drug-selected line and a drug sensitive competitor in mixed-genotype infections.

**Table 1:** Treatment groups and sample sizes.

Infections	Drug treatment				Monitoring days (PI)	Mice per combination	Total # mice
	Dose (mg/kg)	Days (PI)	morning	afternoon			
**Experiment 1: Characterising the resistance phenotype**							
10*6 AS116P(art)*	0, 4*, 16, 32 or 64#	6–10	11am	4pm	3–21	5–7*	60
10*6 AS117P(art)							
10*6 AS109P(s)							
**Experiment 2: Effect of treatment time on drug efficacy**							
10*6 AS109P(s)	32	6–10	9am or 1pm	4pm	3–54	5	20
10*6 AS117P(art)							
**Experiment 3: Drug treatment and within-host competition**							
10*3 AS117P(art)	0,4 or 16	6–8	11am	4pm	3–41	5	45
10*3 AS117P(art)+10*6 AJ (s)							
∼20 AS117P(art)+10*6 AJ(s)							

# 64 mg/kg not included in experiment 1 block A therefore only 5 mice.

*Only used in experiment 1 block A.

PI = post infection.

## Results

### Selection for resistance

**Figure 1:**
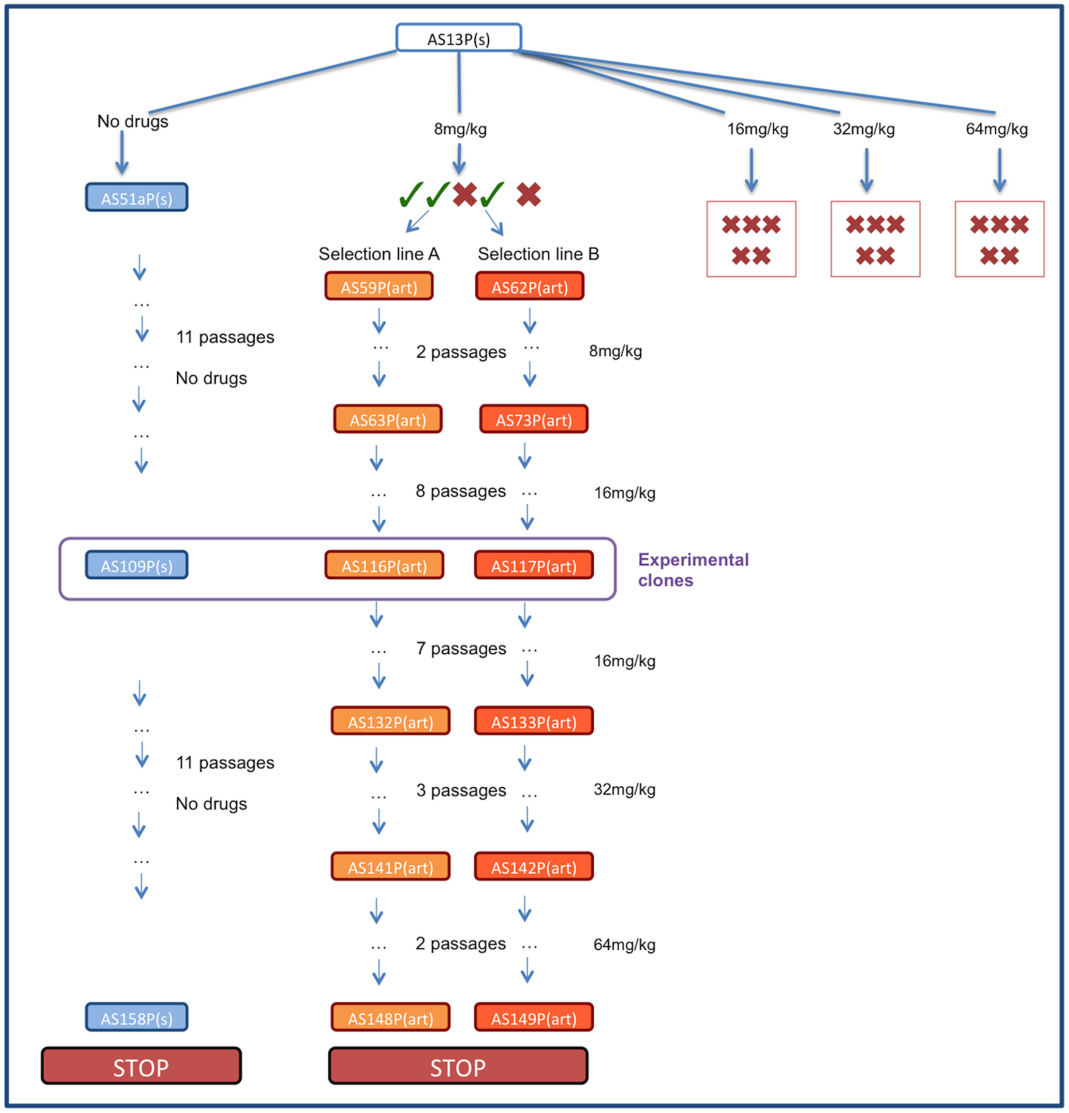
Schematic of selection regime. Infections were initiated with *P. chabaudi* strain AS13P(s) which were treated with 8, 16, 32 or 64 mg/kg of artesunate twice a day for 5 days. No parasites were recovered from any infections treated with 16–32 mg/kg or from 2 infections treated with 8 mg/kg (represented with red crosses). Of the three drug-treated infections with surviving parasites (represented with green ticks) two were used to initiate selection line A and B which were maintained by passaging on surviving parasites under increasing drug pressure. The control line was maintained in parallel without exposure to drugs. The experiments reported here used lines AS116P(art) (experiment 1), AS117P(art) (experiments 1–3) and control line AS109(s) (experiments 1–2).

In C57Bl/6 mice, selection was imposed on *Plasmodium chabaudi* parasites that had never been exposed to drugs (strain AS13P). Initial infections were effectively cleared (no detectible parasites by microscopy for at least 7 days post treatment) with high doses of artesunate (16, 32 or 64 mg/kg twice daily for 4 days; 5 infections [mice] per dose). But, for a lower dose treatment (8 mg/kg twice daily for 4 days), sufficient parasites survived drug treatment in 2 out of 5 infections to establish new infections; these parasites were used to establish our 2 selection lines (Figure 1). The drug selection lines were exposed to increasing drug doses in a step-wise manner (surviving 8 mg/kg for two more passages, then stepping up to 16 mg/kg by the 4^th^ passage) for 11 passages before reaching our experimental, selected lines: AS116P(art) & AS117P(art)). A control line (AS109P(s)) was passaged in parallel through mice without exposure to drugs. We continued selection on all lines for a further 12 passages, stepping up to 32 mg/kg and then 64 mg/kg, to generate at the end parasites in treated lines which survived a dose of 64 mg/kg (figure 1).

### Experiment 1: Characterising the resistance phenotype

In order to test for resistance in our drug-selected lines, we infected mice with 10^6^ parasites from either one of our drug selected lines (AS116P(art) or AS117P(art)) or our control line (AS109P(s)). From day 6 (corresponding to peak parasite density), infections were treated with 4, 16, 32 or 64 mg/kg of artesunate twice a day for 5 days, or left untreated (5–7 mice per parasite and treatment combination). We used two measures of resistance: (1) drug efficacy during treatment, measured as the slope of the parasite clearance curve [47] and its corresponding parasite half-life, i.e. the time taken for 50% of the parasites to be removed by treatment (see materials and methods for details on clearance curve fitting), and (2) parasite recrudescence after treatment, measured as the cumulative parasite density in the week post-treatment.

Experiment 1 was conducted over two experimental blocks. In block A, both of our two replicate selection lines (AS117P(art) and AS116P(art)) were used in addition to the control line (AS109P(s)). There was no significant difference between the two selected lines in either clearance rate under drug pressure (16 mice across 3 drug doses (4, 16 and 32 mg/kg): AS116P(art) vs. AS117P(art) χ^2^_1_ = 0.32, p = 0.58; figure S1) or in the effect of drug pressure on overall infection dynamics (line*treatment*day χ^2^_4_ = 3.91, p = 0.42; figure S2). Therefore, in order to minimise the number of animals used while maximising sample sizes, block B was simplified to use only one selected line, AS117P(art). In addition, the 4 mg/kg treatment group was dropped in block B, allowing us to include a 64 mg/kg treatment group (see table 1), testing the limits of resistance with the highest dose possible without toxic side-effects for the mouse.

**Figure 2:**
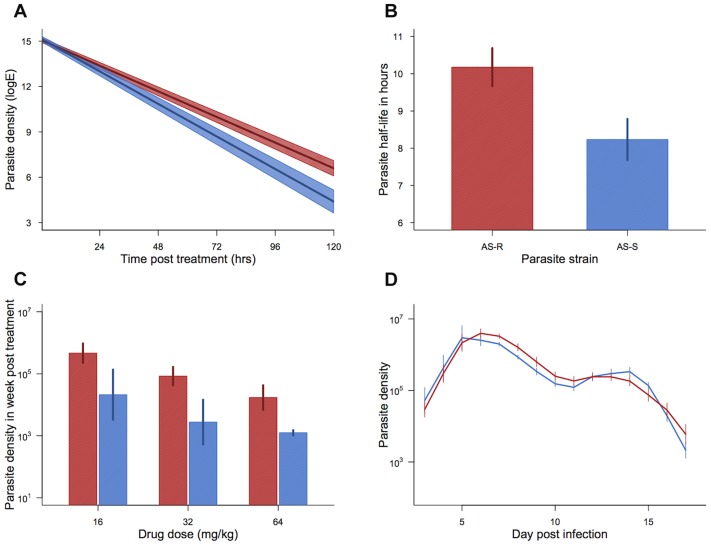
Resistance phenotype and parasite dynamics. Parasite clearance curves (a), half-lives (b), cumulative density in the week post treatment (c), and dynamics in the absence of drugs (d) for drug-selected line (AS117P(art) shown in red) and control line (AS109P(s) shown in blue). Bars (b–d) and shaded area (a) show the standard error of the mean. Mean from 19 infections per line (a & b) and 5–7 infections per combination (c) and 7 infections per line (d). Data from experiment 1.

Across treated infections from both experimental blocks, our drug-selected parasite line cleared at a slower rate, corresponding to a significantly longer half-life than our control line (mean half-life: AS109P(s) = 8.23(±0.55 SEM)hrs; AS117P(art) = 10.18(±0.51 SEM)hrs; χ^2^_1,35_ = 8.78, p = 0.003; figure 2a,b). Parasite half-life was not significantly affected by drug dose (χ^2^_2,33_ = 4.94, p = 0.085), nor an interaction between drug dose and parasite line (Parasite line*drug dose χ^2^_2,31_ = 1.64, p = 0.44). The change in clearance rates and half-lives in our selected line were similar to the resistance phenotype seen in human malaria infections [8]. In keeping with a previous study on *P. falciparum* parasites in culture [40], our drug-selected line also had a more pronounced recrudescence, producing on average more than twice as many parasites in the week following drug treatment than did our control line (F_1,36_ = 8.55, p = 0.006; figure 2c). Although there was a trend for smaller recrudescence under higher drug doses, this effect was borderline non-significant (F_2,35_ = 3.05, p = 0.061), and there was no significant interaction between drug dose and parasite line (F_2,32_ = 0.046, p = 0.96; figure 2c). In the absence of drug treatment, the selected line did not differ in parasite dynamics from the control line, suggesting that our selection regimes had not resulted in changes in the parasite phenotype unrelated to treatment (parasite line*day post infection χ^2^_16,38_ = 21.17, p = 0.17, parasite line χ^2^_1,22_ = 1.10, p = 0.29; Figure 2d). Therefore, our selection regimens generated artesunate-resistant parasites with slower clearance rates and greater magnitude recrudescences after only 11 passages (figure 1).

### Experiment 2: Effect of treatment time on drug efficacy

Decreased drug sensitivity of ring stage parasites has been implicated in slower clearance rates under artemisinin treatment [8], [40], [46]. It was therefore predicted that the timing of treatment, and, therefore, parasite stage for synchronous parasites, would be important for drug efficacy [48]. In order to investigate this hypothesis, in experiment 2, we infected mice with either our resistant (AS117P(art)) or our control line (AS109P(s)) and gave the first treatment of the day (32 mg/kg) either early (9am) or late (1pm). These drug treatment times were chosen to correspond to either a high or low proportion of parasites in early ring stage [49], which was confirmed by microscopy (20 infections per time point: 8.45am 80%(±4.4 SEM) early ring vs. 12.45pm 23%(±2.2 SEM) early ring; Z_1,30_ = −31.79, p<0.001). All mice were given a second drug treatment (32 mg/kg) each day at 4pm.

In support of our earlier experiments, resistant parasites had a significantly longer half-life than our control line (parasite line χ^2^_1,19_ = 8.45, p = 0.009; effect size = 1.63 hrs) but this was not significantly effected by time of day of the first drug treatment (treatment time χ^2^_1,18_ = 0.033, p = 0.86; treatment time*parasite line χ^2^_1,17_ = 0.91, p = 0.35). Although time of treatment had no affect on the half-life of resistant and susceptible lines, it did seem to effect whether or not the resistant line declined immediately following treatment or whether there was a delay of a day between the initiation of drug treatment and a decline in parasite densities. For most cases we observed no lag at all: when the control line received early treatment, 0 out of 5 infections had a lag phase, when the control line received the late treatment, 1 out of 5 infections had a lag phase and when the resistant line received early treatment, 1 out of 5 infections had a lag phase. However, when the resistant line received the late treatment, 4 out of 5 infections had a lag phase. This meant that 2 days after the start of treatment there were more parasites when the resistant parasite received late treatment as compared to early treatment (late treatment = 5.49×10^5^ parasites per µL (±9.3×10^4^ SEM); Early treatment = 3.30×10^5^ per µL (±6.8×10^4^ SEM))

### Transmission implications of slower clearance rates

**Figure 3:**
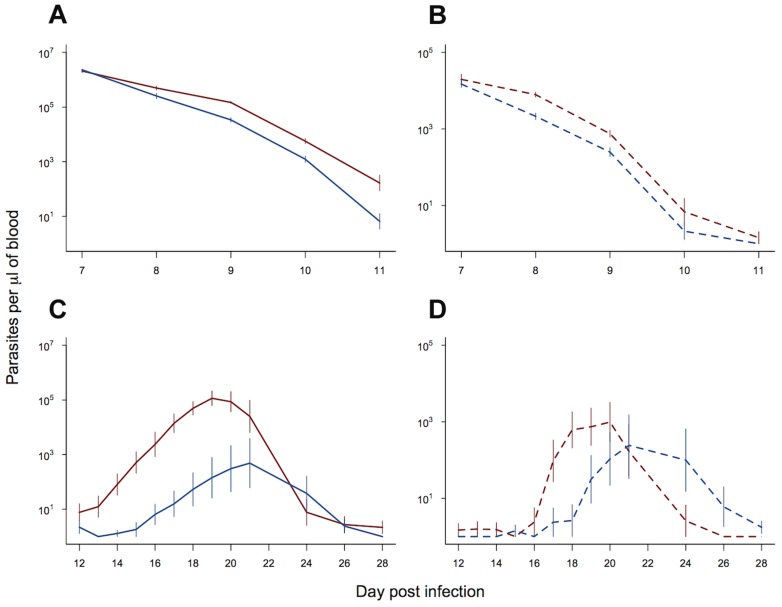
Transmission advantage of selected line. Asexual (solid lines) and gametocyte (dashed lines) density during the period of drug treatment (a & b) and post drug treatment (c & d). Selected line is shown in red and control line in blue. As treatment time had no significant effect on parasite dynamics, means and standard errors are calculated from pooled data (10 replicate infections per line). Bars show the standard error of the mean. Data from experiment 2.

In order to examine the potential fitness benefit of slower clearance times and faster recrudescence in our resistant line, we examined the gametocyte densities in experiment 2. We split our data and examined daily gametocyte counts within two time periods: (i) day 7 to day 11 post infection, corresponding to the period of drug treatment; (ii) day 12 to day 28 post infection, corresponding to the post drug treatment peak in parasite density. Slower clearance rates in our resistant line (figure 3a) resulted in significantly higher gametocyte densities than the control line over the period of drug treatment (parasite line χ^2^_1,18_ = 6.15, p = 0.013; figure 3b). Neither the gametocyte density, nor the rate of decline in gametocytes, was affected by the diurnal timing of drug treatment (treatment time χ^2^_1,17_ = 1.69, p = 0.19; treatment time*parasite line χ^2^_1,16_ = 1.31, p = 0.25). After the completion of drug treatment, both the resistant line and control line infections recrudesced. This recrudescence occurred earlier and was larger for the resistant line, both for asexual parasite densities (day post infection* parasite line χ^2^_12,212_ = 50.66, p<0.0001; figure 3c) and gametocyte densities (day post infection* parasite line χ^2^_12,212_ = 29.60, p = 0.003; figure 3d). Therefore, the drug-selected line produced more gametocytes, both during drug treatment and afterwards during recrudescence.

### Experiment 3: Drug treatment and within-host competition

The effect of drug treatment on our selected lines within mixed infections was examined in experiment 3 by initiating infections with either 10^3^ or ∼20 resistant parasites injected alone or in a mixed inoculum with 10^6^ susceptible competitors. Infections were then left untreated (control group), treated with a low dose of artesunate (4 mg/kg) or treated with a moderate dose of artesunate (16 mg/kg). Drug treatment was given twice a day for 3 days (days 6–8 post infection). This treatment was shorter in duration than in our experiments characterising the resistance phenotype (experiments 1–2), since those experiments were explicitly testing the limits of the resistance phenotype and resistant parasites were at a much lower density at the time of treatment in mixed infections (due to a combination of lower inoculums and competitive suppression). The dynamics of resistant parasites in mixed infections with a susceptible competitor were unaffected by number of parasites in the initial inoculum (10^3^ vs. ∼20 resistant parasites; asexual dynamics χ^2^_1,26_ = 2.04, p = 0.15; gametocyte dynamics χ^2^_1,26_ = 2.61, p = 0.11), and so these treatments were grouped together for further analysis.

**Figure 4:**
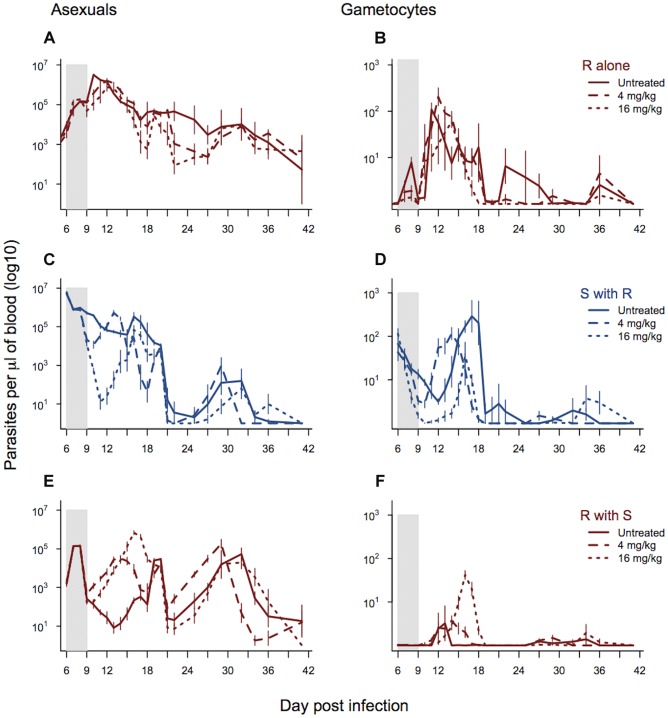
Dynamics of asexual and transmission stage parasites in single and mixed infections under three treatment regimes. Dynamics of asexual parasites (left panels) and gametocytes (right panels). Resistant parasites in single infections shown in panels a–b. Mixed infections shown in panels c–f with susceptible parasite dynamics in blue (c–d) and resistant parasite dynamics in red (e & f). Each line represents a treatment regime with the mean calculated from between 5 and 10 infections. Bars show the standard error of the mean. The shaded area shows the period of drug treatment. Data from experiment 3.

The asexual stage density of drug-selected parasites in the absence of competition was unaffected by drug treatment or dose (drug dose χ^2^_1,12_ = 1.15, p = 0.28; treated vs. untreated χ^2^_1,13_ = 2.39, p = 0.12; figure 4a). Indeed, infections actually continued to grow in the presence of drugs, and at the same rates as they did in untreated infections (figure 4a). In single infections, gametocytes from the resistant line were significantly affected by drug treatment with the higher dose (16 mg/kg) resulting in lower overall gametocyte densities (drug dose χ^2^_1,12_ = 9.69, p = 0.002; figure 4b).

As expected, the parasite dynamics for susceptible parasites in mixed infections were significantly affected by drug treatment and dose, with the highest drug treatment reducing the density of susceptible parasites to the greatest extent. This was the case for both asexual densities (day*drug treatment χ^2^_49,558_ = 256.46, p<0.0001; drug dose χ^2^_24,558,_ = 203.43, p<0.0001; figure 4c) and gametocyte densities (day*drug treatment χ^2^_49,558_ = 321.4, p<0.0001; drug dose χ^2^_24,558_ = 171.4, p<0.0001; figure 4d). Drug treatment, and the corresponding reduction in competition, significantly affected asexual stage resistant parasite dynamics in mixed infections (day*drug treatment χ^2^_49,558_ = 306.06, p<0.0001) and this depended on the drug dose (4 mg/kg vs. 16 mg/kg; χ^2^_24,558_ = 197.85, p<0.0001). Across the whole infection, the highest density of asexual stage resistant parasites occurred in infections treated with the higher drug dose, and the lowest density occurred in the untreated infections (figure 4e and figure S3). This pattern was seen even more strongly in the dynamics of gametocytes (day*drug treatment χ^2^_49,558_ = 334.3, p<0.0001; drug dose χ^2^_24,558_ = 247.6, p<0.0001), where again the highest densities of resistant parasites were seen in the infections treated with the highest drug dose (figure 4f and figure S3). The combination of decreased susceptible parasite densities and increased resistant parasite densities under drug treatment resulted in a dramatic change in the relative abundance of asexual stage resistant parasites within infections with the highest proportion of resistant parasites in the infections treated with the highest dose (drug treatment χ^2^_2,24_ = 30.37, p<0.0001). Similarly, the highest drug treatment led to the greatest relative abundance of resistant line gametocytes (drug treatment χ^2^_2,24_ = 19.17, p<0.0001).

High dose treatment of mixed infections increased the cumulative number of gametocytes produced by the resistant line nearly 7-fold (as compared to densities in low dose treatment) (Fig. 4f and figure S3c). The proportion of mosquitoes infected is a non-linear function of gametocyte concentration; using the empirically derived dose-response curve estimated by Bell *et al.* [41] for *P. chabaudi* in this strain of mouse, this 7-fold difference translates to a 50% increase in the proportion of mosquitoes infected by resistant parasites as a consequence of aggressive drug treatment. In terms of overall transmission of both strains, gametocyte numbers were lower in drug treated infections than untreated controls (drug treated vs. control χ^2^_1,27_ = 9.04, p = 0.01), but there was no additional reduction with a higher dose treatment (drug dose χ^2^_1,27_ = 0.79, p = 0.37).

**Figure 5:**
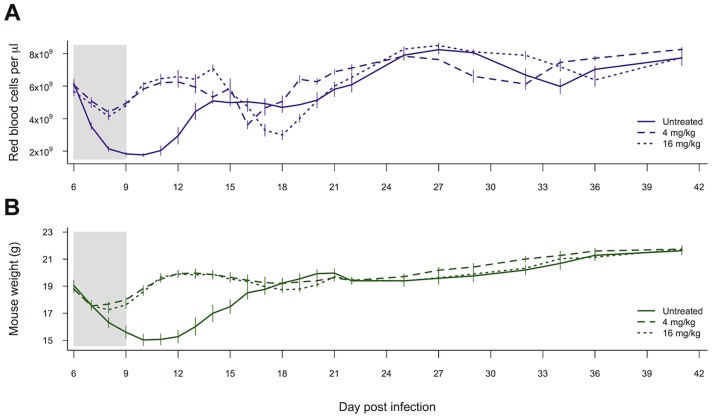
Drug treatment effects on measures of health. Red blood cell density (a) and weight (b) of mice left untreated, given 4 mg/kg of artesunate or given 16 mg/kg of artesunate. Lines represent the mean from between 8 and 10 infections per treatment and bars show the standard error of the mean. The shaded area shows the period of drug treatment. Data from experiment 3.

Both of our measures of host health, i.e., anaemia and weight loss, were affected by whether mice received drugs (red blood cell density: χ^2^_1,28_ = 17.37, p<0.0001; weight χ^2^_1,27_ = 5.83, p = 0.016). However, the higher-dose drug treatment did not result in any additional improvement to health outcomes as compared to the lower drug dose (red blood cell density: χ^2^_1,27_ = 0.18, p = 0.67; weight χ^2^_1,26_ = 0.67, p = 0.41; figure 5).

## Discussion

We have demonstrated that susceptibility to artesunate was reduced within only three passages, with selected parasites breaking through a drug dose of 16 mg/kg that was able to fully clear the ancestral line. After ten passages, selected parasites were cleared more slowly and bounced back more rapidly from all drug doses tested (up to 64 mg/kg twice a day for 5 days: figure 2). The speed at which resistance evolves may be stochastic and likely varies depending on intrinsic differences between parasite strains or species, a question which will be important to address in future work. However our data provides proof of principle that susceptibility to artesunate can be rapidly lost under drug pressure. Consistent with recent reports from *P. falciparum* [50], increasing the drug dose did not increase clearance rates of selected parasites in our study, but, in single infections, gametocyte densities were reduced. The slower clearance rate and longer half-life of our selected lines were qualitatively similar to the rates observed from human malaria infections in South-East Asia [8].

Slower artemisinin drug clearance rates have been well documented in human malaria infections. The spread of slow clearing parasites in South-East Asia suggests that this trait must have a fitness advantage, however the nature of the selective advantage involved has remained unclear. We found that, in addition to slower clearance, resistant parasites recovered more rapidly from treatment, with higher asexual and gametocyte densities in the week post treatment. This could be due to higher numbers of parasites remaining at the end of treatment or faster recovery from a dormant state [38], [40]. Slower clearance rates and faster recovery of parasite densities increased gametocyte density (figures 3 & 4) and, therefore, transmission potential, clearly benefitting the parasite without an obvious cost in the absence of treatment (figure 2d). In *P. falciparum* infections, gametocytes take longer to mature than in *P. chabaudi*, so the timeline may differ, but the general pattern of faster recovery and higher gametocyte densities during recrudescence could provide a significant fitness advantage to resistant parasites.

For the majority of our experimental infections, drug treatment was given at the peak of parasite density, when symptoms become apparent. This timing was chosen because people normally begin treatment only after the onset of symptoms. At that point in a *P. chabaudi* infection, parasite numbers are also beginning to be negatively impacted by host immune responses and resource limitation, so that our estimated clearance rates are likely to have captured parasite death due to a combination of a deteriorating within-host environment as well as drug action. The potential effect of host environment on parasite growth can be seen in our single-infection controls for the competition experiment (figure 4a). These infections were initiated with a smaller inoculum (∼20 and 1000 vs. 10^6^ parasites in experiments 1–3), which delayed the peak in parasite density. This meant that untreated infections were still in growth phase when drugs were administered to the treatment group; in this case, parasite densities continued to rise in the presence of drugs. This observation demonstrates that our selected line was largely resistant to artesunate, and it agrees with previous data suggesting that clearance rates estimates can be highly dependent on the within-host environment [51].

The mode of action for artemisinin-based drugs, and the mechanism by which parasites lose susceptibility, remains unclear. Models of *P. falciparum* infection dynamics from areas with variable clearance rates suggest that susceptibility to artemisinin may be parasite stage specific, with resistance developing predominantly in the ring stage parasite [46], [52]. We found no evidence that targeting different cohorts of parasites influenced the efficacy of our treatment as measured by parasite half-life. There was, however, a trend suggesting that treatment time, and presumably parasite stage, influenced the likelihood of a lag between the initiation of drug treatment and the beginning of parasite clearance.

The majority of malaria infections consist of multiple genotypes, so that when resistance is rare, as it necessarily is when it first arises, the fitness benefits and costs of resistance will largely depend on interactions with susceptible competitors. Aggressive drug treatment of malaria infections with pyrimethamine can result in ‘competitive release’ of resistant parasites by removing susceptible competitors, while resistant parasites remain unaffected [33]–[36], [41]. Until now, it was unclear whether competitive release would occur with artemisinins, particularly where the resistance phenotype is associated with slower clearance times [18]–[20]. In our experiments, drug-selected parasites with slower clearance rates experienced significant competitive release after drug treatment (figure 4). Furthermore, the strength of this competitive release was dose-dependent, with the highest densities of selected parasites occurring in the most strongly treated infections (figure 4e and figure S3). This was particularly striking for transmission stages, where competitive release after drug treatment allowed resistant parasites to dominate the pool of gametocytes (figure 4f and figure s3). The extent of competitive suppression in untreated infections depends on the identity of the competitors [53]–[54]. This means that the magnitude of the increase in resistant parasites following the removal of competitors by chemotherapy will likely also depend on the clones involved. But drug treatment will always disproportionately kill the most susceptible strains, so that competitive release will occur. As our new data show, this is dependent on artesunate dose, just as it was dependent on pyrimethamine dose in our previous work [34], [36]. Further work evaluating competitive release across a wide range of clones, drugs, initial conditions and host variation is certainly warranted to determine whether aggressive chemotherapy provides a strong selective advantage for resistant parasites in general. In our data, a lower drug treatment (6 doses of 4 mg/kg) improved the health of host mice by reducing both weight loss and anaemia relative to no-treatment controls, but there was no additional benefit to treating more aggressively (6 doses of 16 mg/kg). In fact, our higher dose treatment led to a slightly lower dip in red blood cell density during the post treatment recrudescence (figure 5a). This supports the suggestion that less aggressive drug treatment may, in some cases, be able to limit the selective advantage for resistant parasites, without compromising health outcomes [26], [36] (but see [55]–[57] for discussion of this approach).

Although aggressive chemotherapy resulted in a strong selective advantage for resistant parasites (figure 4), none of our initial infections survived drug doses higher than 8 mg/kg (figure 1). It is possible that a larger number of infections being treated when parasite densities are maximal would have resulted in some breakthrough from higher drug doses (see [58], [59] for examples of resistance arising from selection with high drug doses), but given our sample sizes, aggressive chemotherapy was successful at preventing *de novo* resistance from arising. In contrast, parasites exposed to a step-wise increase in drug doses displayed significant increases in resistance, surviving 8 times the drug dose they were exposed to at the start of the selection regime (figure 1). This demonstrates the double-edged sword at the heart of the resistance management dilemma: aggressive chemotherapy may prevent resistance from emerging in the first place, but it also provides the strongest selection for resistant parasites once they are present in an infection [26]. Moderate chemotherapy, on the other hand, could increase the chances of a partially resistant mutant surviving drug treatment, but will be a weaker selective force on existing resistance mutants. Which regimens most effectively slow the spread of resistance in the real world without compromising patient health is difficult to predict. Many factors will be important [26], not least the number of mutations required to confer resistance, the cost of resistance in the absence of treatment, the within-host ecology of infections and the clinical consequences of treating infections which do, or do not, contain resistant parasites. The successful use of combination therapy is also likely to reduce the probability of resistant mutants surviving within an infection and reaching transmissible densities [2], [38], [60]–[61]. Further experiments to elucidate the relative contributions of *de novo* resistance versus the spread of existing resistant strains, and the impact of partner drugs, will be essential for developing evidence-based resistant management programs [26].

In conclusion, we have shown that artesunate treatment of malaria-infected mice can rapidly select for slower clearance times resembling those in human malaria cases. Furthermore, our selected parasite line with slower clearance rates and faster recovery from drug treatment had a selective advantage over a drug susceptible competitor via increased transmission potential in both single and mixed strain infections. Finally, we demonstrated that a more aggressive drug dose could, in at least some cases, increase the selection for resistant parasites without providing an additional health benefit. Caution is obviously required in generalising results from a model system. The speed at which the slow-clearance phenotype spreads though parasite populations will be determined by many interacting factors, including vector ecology, chemotherapy, natural immunity and parasite ecology within the vector and the human host. Competitive interactions between co-infecting clones cannot be formally demonstrated in human infections because untreated controls are required, but many lines of indirect evidence suggests competition also occurs in human malaria infections [27], [29], [62]–[69]. Nonetheless, the mechanisms of competition and of resistance, and therefore the relative costs and benefits of resistance, likely differ in magnitude between human and rodent malaria parasites. Thus our findings highlight that consideration should be given to developing evidence-based treatment regimes, with the aim of maximising both health gains and resistance management.

## Materials and Methods

### Parasites and hosts

Both the parental AS *P. chabaudi* strain (AS13P) used in the selection regimes and the susceptible AJ strain parasites used in the competition experiment (experiment 3) were originally collected from thicket rats (*Thamnomys rutilans*) in the Congo [70], maintained as part of the WHO Registry of Standard Malaria Parasites (The University of Edinburgh), transported to Penn State University and stored in liquid nitrogen. Mice in our experiments were 6–10 week old female C57Bl/6. All mice received 0.05% PABA-supplemented drinking water to enhance parasite growth [71]. The mice were fed on Laboratory Rodent Diet 5001 (LabDiet; PMI Nutrition International, Brentwood, MO, USA) and were kept on a 12∶12 light∶dark cycle. For sample sizes and treatment groups across experiments see table 1.

### Selection for resistance

Infections were initiated in 20 mice via intraperitoneal (IP) injection of 10^6^ susceptible parasites (AS13P) and treated with 8, 16, or 32 mg/kg artesunate on days 2–6 post-infection, or with 64 mg/kg artesunate on days 6–10 post infection (5 mice per dose). Drug treatment was given twice a day as an IP injection of artesunate (Sigma-Aldrich) dissolved in sterile water with the dose adjusted for the weight of the mouse at approximately 11am and 4pm. Pilot work suggested that twice-daily treatments were more effective. *P. chabaudi* has a 24 hour life-cycle, meaning that twice daily treatment is equivalent to daily treatment of *P. falciparum*, which has a 48 hour life cycle.

Previous studies selecting for resistance to artemisinins have varied in their precise selection regimes but have, in general, treated early in the infection [56]–[57], [72]–[74]. The treatment days for the 8–32 mg/kg treatment groups were chosen to fit more closely with these previous methods, while the 64 mg/kg group parasites were allowed to grow up to peak density at day 6 post infection to increase the parasite pool from which to select a resistant mutant. Additionally, our selection regime differed from a previously published selection for artemisinin resistance [74] in that we used fully-susceptible ancestral parasites rather than lines resistant to other anti-malarial drugs as a starting point [74], [ but see 39].

Thin blood smears were taken from all infections from two days after the final drug treatment and examined for the presence of parasites. In the 64 mg/kg group, blood was additionally passaged on to naïve mice at two-day intervals (days 15–25 post infection) to test if parasites were present below the detection threshold. No parasites were detected in mice treated with the three higher doses, and no infections established in naïve mice. In the 8 mg/kg group, three out of five mice had microscopically-detectable recrudescence, two of which reached sufficient densities to passage 10^6^ parasites to new mice, starting parallel selection lines (selection lines A & B; five infections per line were initiated from each of the two donors; see figure 1). The same 8 mg/kg dosing regime was then repeated on the new infections; all five mice had parasite recrudescence in line A, and four out of the five mice had recrudescence in line B. Subsequently, all further passages (typically between day 10 and 14 post infection, when ≥2% of host red blood cells were infected) were done with 10^6^ parasites to two mice, always with drugs given on day 2 post infection, then twice daily for 5 consecutive days. For each passage, the mouse with the highest parasitaemia from each replicate was chosen. After three rounds of selection at 8 mg/kg, passages were performed in a similar way as described above, but with double the drug dose (16 mg/kg). Both lines were passaged eight times at this dose to arrive at the experimental clones (AS116P(art) and AS117P(art)). Subsequently, we continued our selection for another 7 passages at 16 mg/kg, 3 passages at 32 mg/kg, followed by two passages at 64 mg/kg, to derive the final selected clones AS148P(art) and AS149P(art).

From the same ancestral clone as the drug-selected lines (AS13P), an untreated control line was evolved. Two mice were inoculated with 10^6^ parasites and then the mouse with the highest parasitemia to two new mice at 10^6^ at day 7 post infection. After twelve passages, this led to experimental control clone AS109P(s). Note that this control clone was passaged one more time than the experimental line. At each passage stage, parasites were frozen down for storage and all parasites are maintained on liquid nitrogen at The Pennsylvania State University.

### Experiment 1: Characterising the resistance phenotype

Mice were infected as previously described with 10^6^ parasites of AS116P(art), AS117P(art) or AS109P(s) (the control line). Infections were either left untreated or treated with artesunate (4, 16, 32 mg/kg in block A & 16, 32 or 64 mg/kg in block B) twice a day for 5 days (∼11am and ∼4pm on days 6–10 post infection). Infections were monitored daily from day 3–21 post infection. During sampling, mouse weight and red blood cell density (by Flow Cytometry, Beckman Coulter Counter; [see 75] were measured, and thin blood smears were taken. Additionally, 5 µL of blood was taken to estimate total parasite densities [see 34].

Parasite clearance rate was calculated by fitting the slope of the linear decline in parasite density over time (on a natural log scale) during the period of drug treatment [42], [47], [76]. For some infections (9/84 across all experiments), there was a lag in the clearance, during which the density of parasites continued to increase for one day after treatment but then declined. This lag is also seen in some human infections [47]. For these cases, the slope was fitted from day 7 rather than day 6 [76]. A linear model provided a good fit for our data (mean R^2^ = 0.93±0.0047 S.E.), and so these clearance slopes were used to calculate the parasite half-life during drug treatment for each infection (half-life in hours = (natural log of 2/absolute clearance slope)×24)).

### Experiment 2: Effect of treatment time on drug efficacy

In experiment 2 infections were initiated with 10^6^ parasites of either the drug selected line (AS117P(art)) or the control line (AS109P(s)) treated with 32 mg/kg twice daily for 5 days (days 6–10 post infection). Half of our mice received the first treatment of the day at 9am and half at 1pm (5 mice per line per treatment time). All mice were given a second dose of drug each day at 4pm. Thin blood smears were taken from all mice at 8.45am and 12.45pm (15 minutes prior to drug treatment) in order to check the stage of parasites exposed to drugs. Slides were fixed in methanol, and stained with Giemsa. To establish the proportion of early stage rings present in infections, slides from day 5 post-infection were examined under the microscope and a minimum of 100 parasites per infection (10 mice per parasite line) staged for each time point. Staging was carried out on day 5 infections to avoid the possibility of drugs differentially killing some parasite stages.

Infections were monitored daily from day 3 to day 21 post infection and then three times a week until day 54 post infection. In addition to the measurements made in experiment 1, 10 µL of blood a day was taken to estimate gametocytes by quantitative PCR [see 34].

### Experiment 3: Drug treatment and within-host competition

Mixed infections were initiated with either 10^3^ or ∼20 AS117P(art) parasites inoculated at the same time as 10^6^ parasites of a susceptible competitor (*P.chabaudi* strain AJ). Single infection controls were initiated with 10^3^ AS117P(art) parasites. Infections were then either left untreated, treated with a low dose of artesunate (4 mg/kg twice a day for 3 days), or treated with a high dose of artesunate (16 mg/kg twice a day for 3 days). Infections were monitored daily for mouse weight, red blood cell density, asexual stage parasites and gametocytes from day 3–21 post infection. Further monitoring of infections occurred three times a week until day 41. Genotype-specific qrtPCR allowed us to monitor parasite densities from our drug-selected parasite line and a susceptible competitor line independently (for both total parasite and gametocyte densities) over the full experiment [77].

### Statistical analysis

All statistical tests were carried out using R version 2.14.1 (http://www.R-project.org). Parasite half-lives (log transformed) and the cumulative density in the week post treatment were analysed using general linear models (Gaussian error structure). The proportion of ring stage parasites was analysed with a general linear model with a binomial error structure. Asexual densities (log10 transformed), gametocyte densities (log10 transformed) and the proportion of resistant parasites over time (arcsine square root transformed) were analysed using linear mixed effect models with mouse (nested within block for experiment 1) as random effects. As is common with repeated-measure parasite data [78], there was significant temporal autocorrelation within our dataset. We therefore fitted a corAR1 autocorrelation structure with mouse nested within day into our models [78], [79]. For all analyses over multiple days of infection, day was included as a factor to account for non-linear infection dynamics over time. We followed model simplification by sequentially dropping the least significant term and comparing the change in deviance with and without the term to χ^2^ distributions, until the minimal adequate model was reached. Degrees of freedom correspond to the difference in the number of terms in the model.

### Ethics statement

This study was carried out in strict accordance with the recommendations in the Guide for the Care and Use of Laboratory Animals of the National Institutes of Health. The protocol was approved by the Animal Care and Use Committee of the Pennsylvania State University (Permit Number: 27452).

## Supporting Information

Figure S1 **Comparison of parasite clearance curves for the two replicate selection lines AS117P(art) and AS116P(art).** Mean clearance curves for AS117P(art) (dark red) and AS116P(art) (orange). Dashed lines show the standard error around the mean. Mean clearance rate taken from across 3 drug doses (4, 16, or 32 mg/kg). Data from Experiment 1 block A.
(TIFF)

Figure S2 **Comparison of parasite dynamics for the two replicate selection lines AS117P(art) and AS116P(art).** Parasite dynamics for AS117P(art) (dark red) and AS116P(art) (orange) in untreated infections and in infections treated with 4, 16, or 32 mg/kg of Artesunate. Shaded area indicates the period of drug treatment. Data from Experiment 1 block A.
(TIFF)

Figure S3 **Drug treatment and within-host competition: cumulative parasite densities from the start of drug treatment.** Cumulative total parasite density (A–B) and cumulative total gametocyte density (C–D) after the start of drug treatment (day 6–41 post infection). Drug selected line (AS117P(art)) is shown in red (A & C) and susceptible competitor (AJ) in blue (B & D). Density of the drug-selected line significantly increases with drug dose for both asexuals (F_2,24_ = 20.12, p<0.0001) and gametocytes (F_2,24_ = 9.50, p<0.001). For the susceptible competitor there is a non-significant negative relationship with drug dose for asexual density (F_2,24_ = 0.64, p = 0.54) and a significant negative relationship for gametocytes (F_2,24_ = 4.36, p = 0.024). Data are taken from experiment 3 and show summary statistics for the same patterns shown in figure 4.
(TIFF)
