# Correction: Rapid Response to Selection, Competitive Release and Increased Transmission Potential of Artesunate-Selected *Plasmodium chabaudi* Malaria Parasites

**DOI:** 10.1371/journal.ppat.1004184

**Published:** 2014-05-13

**Authors:** The PLOS Pathogens Staff

The grant funding from J. Juliano is missing from the original article. The correct funding statement is as follows:

This study was funded by the Institute of General Medical Science (R01 GM089932 to AFR) and Institute of Allergy and Infectious Disease (R01 AI089819 to J. Juliano, University of North Carolina) of the National Institutes of Health. The funders had no role in study design, data collection and analysis, decision to publish or the preparation of the manuscript.
