# Supplementary figures and images for: Rapid Response to Selection, Competitive Release and Increased Transmission Potential of Artesunate-Selected Plasmodium chabaudi Malaria Parasites
Source: PLoS Pathog. 2014 Apr 24;10(4):e1004019. doi: 10.1371/journal.ppat.1004019 (PMC3999151; doi:10.1371/journal.ppat.1004019)

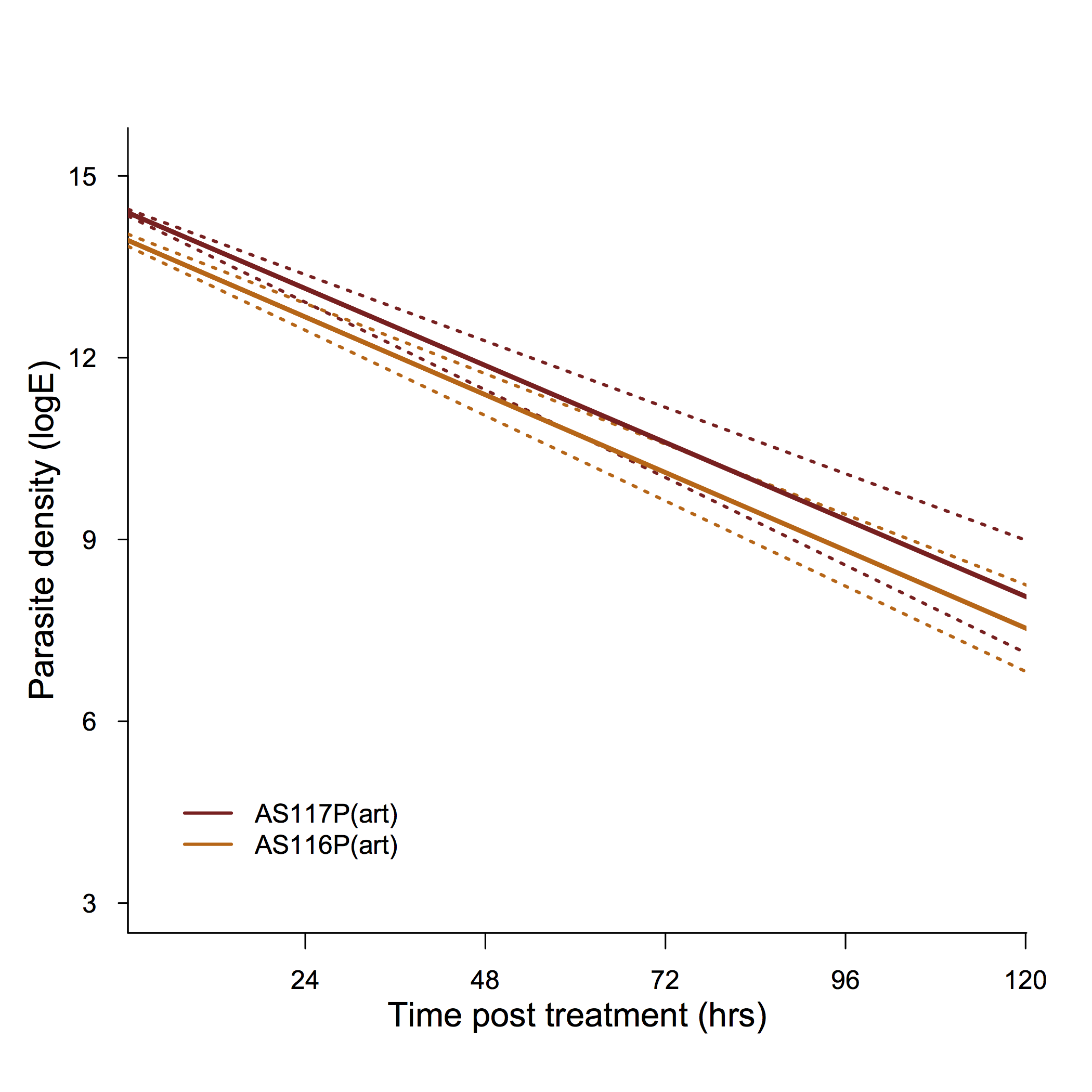

Supplement: Figure S1 — Comparison of parasite clearance curves for the two replicate selection lines AS117P(art) and AS116P(art). Mean clearance curves for AS117P(art) (dark red) and AS116P(art) (orange). Dashed lines show the standard error around the mean. Mean clearance rate taken from across 3 drug doses (4, 16, or 32 mg/kg). Data from Experiment 1 block A. (TIFF) [file ppat.1004019.s001.tiff]

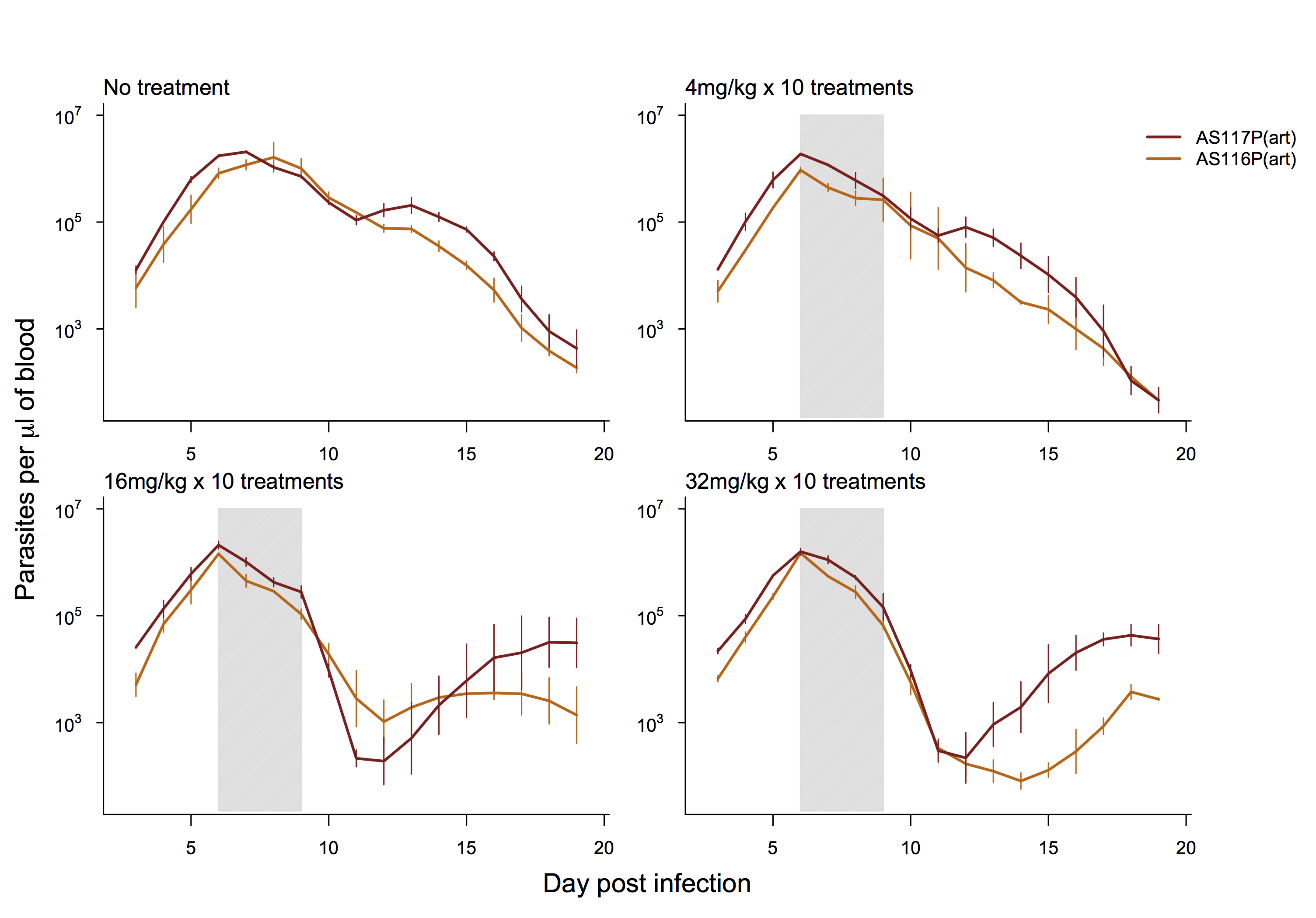

Supplement: Figure S2 — Comparison of parasite dynamics for the two replicate selection lines AS117P(art) and AS116P(art). Parasite dynamics for AS117P(art) (dark red) and AS116P(art) (orange) in untreated infections and in infections treated with 4, 16, or 32 mg/kg of Artesunate. Shaded area indicates the period of drug treatment. Data from Experiment 1 block A. (TIFF) [file ppat.1004019.s002.tiff]

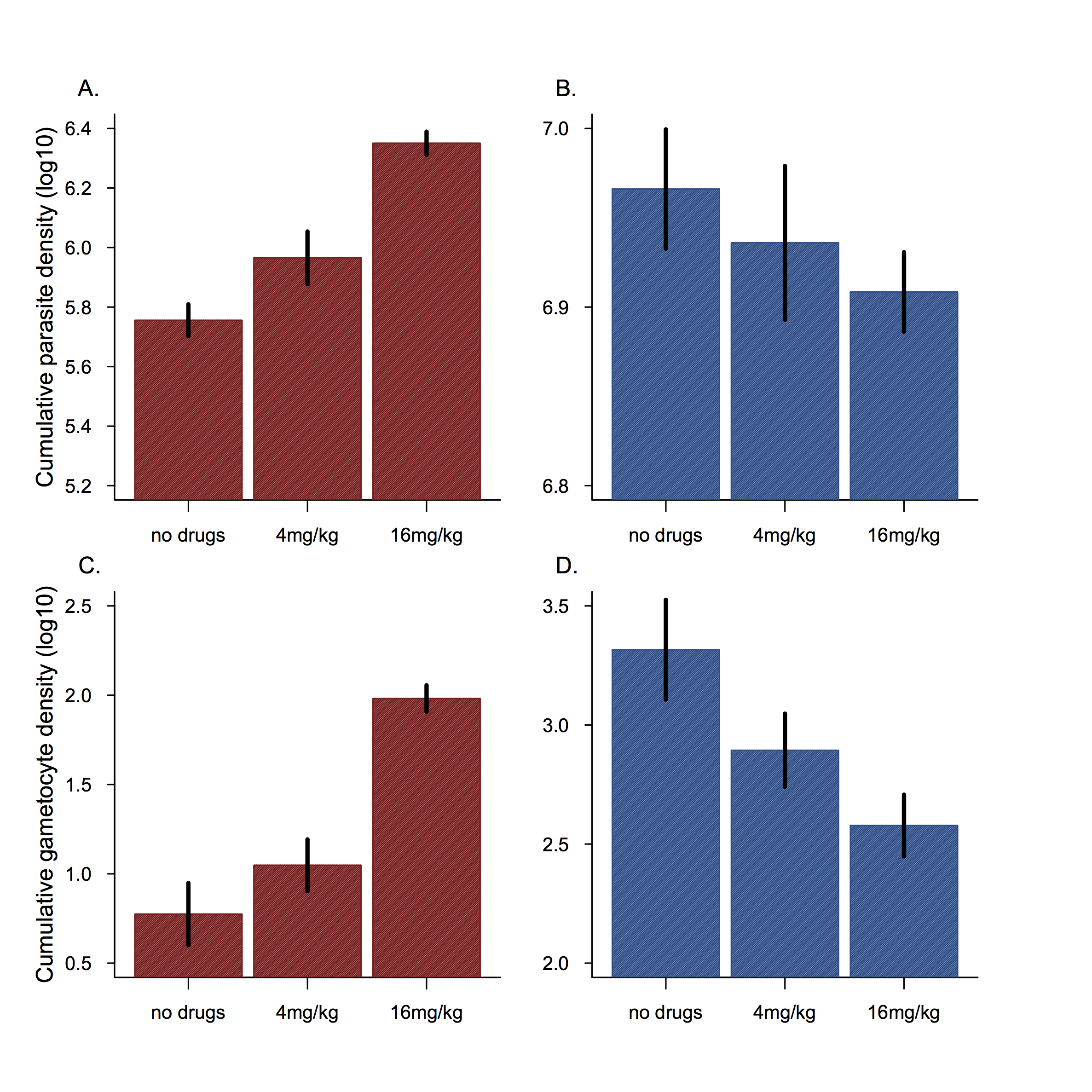

Supplement: Figure S3 — Drug treatment and within-host competition: cumulative parasite densities from the start of drug treatment. Cumulative total parasite density (A–B) and cumulative total gametocyte density (C–D) after the start of drug treatment (day 6–41 post infection). Drug selected line (AS117P(art)) is shown in red (A & C) and susceptible competitor (AJ) in blue (B & D). Density of the drug-selected line significantly increases with drug dose for both asexuals (F2,24 = 20.12, p<0.0001) and gametocytes (F2,24 = 9.50, p<0.001). For the susceptible competitor there is a non-significant negative relationship with drug dose for asexual density (F2,24 = 0.64, p = 0.54) and a significant negative relationship for gametocytes (F2,24 = 4.36, p = 0.024). Data are taken from experiment 3 and show summary statistics for the same patterns shown in figure 4. (TIFF) [file ppat.1004019.s003.tiff]
